# Effects of Full Body Exergaming in Virtual Reality on Cardiovascular and Muscular Parameters: Cross-Sectional Experiment

**DOI:** 10.2196/12324

**Published:** 2019-08-28

**Authors:** Boris Feodoroff, Ippokratis Konstantinidis, Ingo Froböse

**Affiliations:** 1 Institute of Movement Therapy and Movement-Oriented Prevention and Rehabilitation German Sport University Cologne Cologne Germany

**Keywords:** exergaming, gamification, Immersive virtual reality, exercise, cybersickness, flight simulation

## Abstract

**Background:**

In recent years, many studies have associated sedentary behavior in front of screens with health problems in infants, children, and adolescents. Yet options for exergaming—playing video games that require rigorous physical exercise—seem to fall short of the physical activity levels recommended by the World Health Organization.

**Objective:**

The purpose of this study was to investigate the effect of a fully immersive virtual reality (VR)-based training system on cardiovascular and muscular parameters of young adults.

**Methods:**

A cross-sectional experiment design was used to analyze muscle activity (surface electromyography), heart rate, perceived exertion (RPE), cybersickness symptoms, perceived workload, and physical activity enjoyment (PACES) in 33 participants performing two 5-minute flights on a new training device.

**Results:**

Participants’ performance of the planking position required to play the game resulted in moderate aerobic intensity (108 [SD 18.69] bpm). Due to the mainly isometric contraction of the dorsal muscle chain (with a mean activation between 20.6% [SD 10.57] and 26.7% [SD 17.39] maximum voluntary isometric contraction), participants described the exercise as a moderate to vigorous activity (RPE 14.6 [SD 1.82]). The majority reported that they enjoyed the exercise (PACES 3.74 [SD 0.16]). However, six participants had to drop out because of cybersickness symptoms and two because of muscle pain due to prior injuries.

**Conclusions:**

Our findings suggest that fully immersive VR training systems can contribute to muscle-strengthening activities for healthy users. However, the dropout rate highlights the need for technological improvements in both software and hardware. In prevention and therapy, movement quality is a fundamental part of providing effective resistance training that benefits health. Exergaming on a regular basis has the potential to develop strong muscles and a healthy back. It is essential that future VR-based training systems take into account the recommendations of sport and exercise science.

## Introduction

### Background

There is a growing body of evidence suggesting that a high level of sedentary behavior can harm human health [1-5]. Despite the advantages that come with access to information and rapid communication, sitting in front of screens for hours at a time has been associated with health and psychological problems in infants, children, and adolescents [6]. Screen time and total sitting time have been the most studied exposure variables in epidemiological research [7]. Of these, television viewing has been proposed to be the most deleterious [8-11]. Recently, this field of interest has expanded to examine the impact of computer and video game time on health [12].

The World Health Organization (WHO) has identified the appearance of negative symptoms due to excessive digital gaming as a disorder. Sedentary behavior spent in front of screens has been reported to increase the risk of obesity, high-density lipoprotein dysfunction, and high blood pressure, which are also major risk factors for cardiovascular morbidity [13-15]. Adolescent boys who spend a great deal of time playing video games have been found to have lower bone mineral densities than average [16,17]. Sedentary behavior spent in front of screens is a primary contributor to decreasing physical activity among youths [18-20]. The promotion of exergaming seems to be a promising way to counteract this trend and its negative effects.

### Exergaming

The portmanteau word exergaming combines exercise and gaming [21]. The term exergame has many definitions, reflecting the diverse approaches undertaken by various research communities during the past 20 years. Health researchers have introduced several terms similar to exergaming in describing the promotion of physical activity or interactivity during video gaming: physical gaming, exertainment, active video games, and active video training [21]. Bogost [22] refers to exergaming as “the combination of exercise and video games,”—the term video gaming is defined as “the process of gaming in any digital device” [22], but the term exercise allows for a variety of interpretations.

Exercise can refer to the process of becoming more skilled in a set of actions, without necessarily specifying the degree of body movement involved [23]. In this respect, exergaming can refer to the process of training reaction times in a solely sedentary setting, provided that it is planned and structured. Professional players of multiplayer online battle arena games optimize, train, and exercise their eye-hand coordination skills.

Exercise can also describe physical activity involving body movements that do not promote physiological adaptation mechanisms or increase a particular skill level [23]. For instance, Pokémon Go promotes body movement by forcing users to change their physical location. Positive beneficial health behaviors such as a higher level of physical activity, more socialization, and better mood have been associated with Pokémon Go [24].

Using the term exercise interchangeably with physical activity or even physical fitness can, therefore, be misleading when referring to exergaming systems. The proposed definition by Oh and Yang [21] describes exergaming as “playing exergames or any video games that require physical exertion or movements that are more than sedentary activities and also include strength, balance, and flexibility activities.” Immersive virtual reality (VR)-based exergaming systems such as VR Boxing and Fastest Fist try to bridge the gap between plain body movements and the planned, structured, and repetitive elements of a training session.

Physical exercise includes an effective stimulation of the muscle adaptation mechanism. WHO recommends that adults between the ages of 18 and 64 years should do at least 150 minutes of moderate-intensity aerobic physical activity throughout the week, at least 75 minutes of vigorous-intensity aerobic physical activity throughout the week, or an equivalent combination of moderate- and vigorous-intensity activity. Muscle-strengthening activities should be done for major muscle groups on two or more days per week [25].

Different muscle contraction types can produce different gains in muscle strength and power [26]. The most common contraction types in everyday life are static isometric contractions (holding an object against gravity) and eccentric and concentric contractions, which in combination produce a dynamic motion (such as lifting and lowering an object). The technical capabilities of VR-based motion capture systems can motivate the performance of isometric contractions, for example, by getting users to execute a perfect plank, an exercise known mostly for its ability to improve balance and strengthen core muscles.

### Plank Exercise

In 2009, lower back pain ranked third among the most common syndromes and illnesses [27]. Core strength training is the most effective way to help alleviate the symptoms of chronic lower back pain [28]. In the exercise known as the plank, the body is fully stretched with its weight supported entirely by the tips of the toes and the forearms. This primarily activates the core muscles (*musculus erector spinae* and *m. rectus abdominis*) together with the deltoids. Varieties of this strengthening exercise have been introduced with and without the use of external devices [29-31].

### Study Objectives

To the best of our knowledge, this is the first qualitative study to examine the muscular training potential of an immersive VR-based full-body exergaming system. The study also analyzed the impact of exergaming on the cardiovascular system and its potential to provide effective endurance training. Understanding its impact on participants’ perceived levels of motion sickness, cognitive load, and overall enjoyment could provide insight into the potential and utility of similar approaches.

## Methods

### Participants

**Figure 1:**
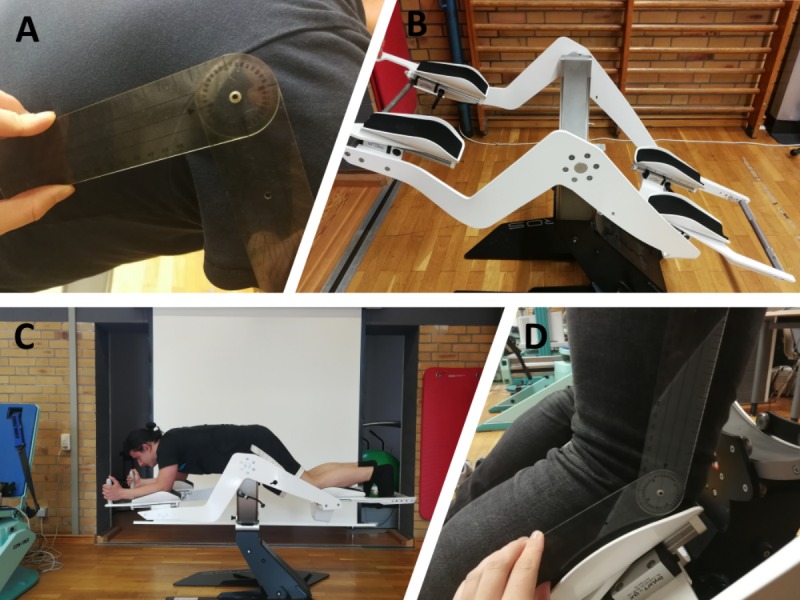
(A) Torso-arm angle measurement using protractor, (B) Icaros device, (C) participant being familiarized with the device, (D) upper-to-lower limb angle measurement using protractor.

Thirty-three participants (mean age 23.90 [SD 4.58] years) were recruited via social media platforms, email chains, and flyers. The study protocol was approved by the ethics committee of the German Sport University Cologne. Most of the participants were students at the German Sport University Cologne. Data collection occurred between June and August 2017. Participant requirements were defined to ensure that factors such as sex, age, and physical condition would not bias the data:

- Male participants aged younger than 30 years: due to hormonal changes, women tend to be more susceptible to motion sickness than men [32].
- Athletic body: body mass index correlates with body fat percentage, which can influence the signal quality of surface electromyography (sEMG). To get a valid sEMG signal, participants had to have a BMI <25 kg/m^2^, indicating a low body fat percentage.
- Height between 170 and 190 cm: the Icaros device (Figure 1) permits configuration of the distance between the foot holder and arm rest. This distance influences muscle activation of the abdominal and back areas. Therefore, a standardized configuration of the upper torso-to-arm and upper-to-lower-body angle was selected. In addition, the body’s center of gravity had to be in line with the device’s zero balance point. Based on these requirements, the device’s properties allowed a standardized configuration for body heights between 170 and 190 cm.

### Instrumentation

The experiment protocol required a variety of different systems to be used simultaneously and synchronized for data analysis.

#### Icaros System

The Icaros VR fitness machine and flight simulator (Icaros GmbH) consists of a device and an attached gyro sensor (Figure 1). The gyro sensor connects via Bluetooth with a smartphone and provides angular velocity data. Body position changes on the sagittal and longitudinal axes of the device are independent of the visual stimuli, allowing a deeper immersion in the VR world through a combination of visual, acoustic, and proprioception sensors.

The device can be rotated around the pivot point with a range of motion between –35° and 35° for roll and –45° to 45° for pitch on the vertical and sagittal axes, respectively.

#### Head-Mounted Device

The VR system employed the Gear VR headset version SM-R322 (Samsung Electronics) and gamepad for setting in-game options. The Icaros software was installed on a Galaxy S6 (Samsung Electronics) smartphone running Android 6.01. The integrated smartphone accelerometer and gyroscope paired with the head-mounted display allowed users to interact with the visual stimuli in virtual reality while maintaining their body position on the Icaros.

#### Heart Rate Monitor

Participant heart rates were measured continuously using the RS800 heart rate monitor (Polar Electro).

#### Muscle Activity

Muscle activity was measured based on sEMG using the TeleMyo 2400T G2 (Noraxon USA).

#### Questionnaires

The study employed questionnaires measuring sociodemographic and anthropometric data. The Simulator Sickness Questionnaire (SSQ) was used to assess perceived motion sickness and cybersickness symptoms during the flights. Enjoyment of the flight sessions was measured using the Physical Activity Enjoyment Scale (PACES). Results are based on the mean average of the 16-item modified version of the questionnaire ranging from a scale of 1 (strongly disagree) to 5 (strongly agree). A high score indicates a high level of physical activity enjoyment [33]. The Borg scale [34] was used to measure the rate of perceived exertion (RPE). This scale has a high degree of validity for endurance training and physical activity [35,36]. The perceived mental, physical, and emotional demands on participants during the VR experience were assessed using the NASA Task Load Index (NASA-TLX) questionnaire [37]. Five of the six dimensions (frustration, effort, mental demand, physical demand, and temporal demand) ranged from 0 (low) to 100 (high). The performance dimension ranged from 0 (good) to 100 (bad).

### Experimental Protocol

All participants were informed about the aim of the study and provided with a written description of the procedure. Participants completed questionnaires collecting sociodemographic and anthropometric data (Table 1). The heart rate monitor system was then configured and participants performed the deep breathing technique [38] for measuring their resting heart rates while seated.

For each test, participant’s height was measured, and the distance between the Icaros arm and foot holders was configured to align the body’s center of gravity with the pitch axis’ zero degree point. The upper-to-lower limb and torso-to-arm angle were configured to 135° and 90°, respectively (seen in Figure 1A and D).

**Figure 2:**
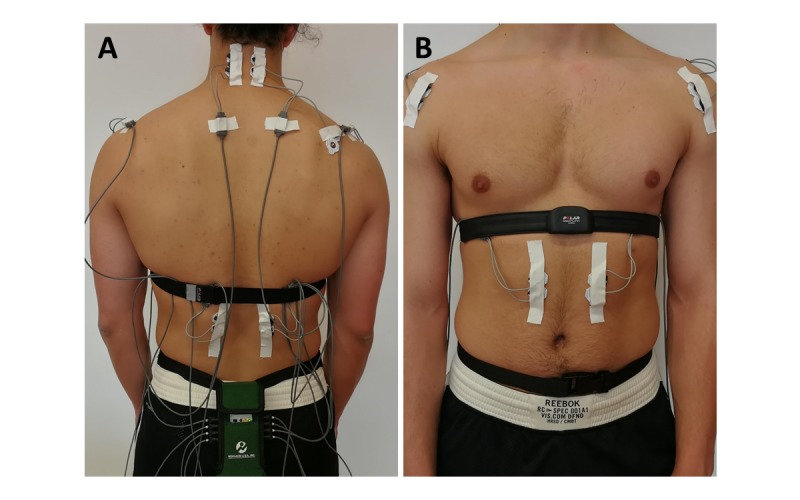
Positioning of electromyography electrodes, hardware, and software.

**Figure 3:**
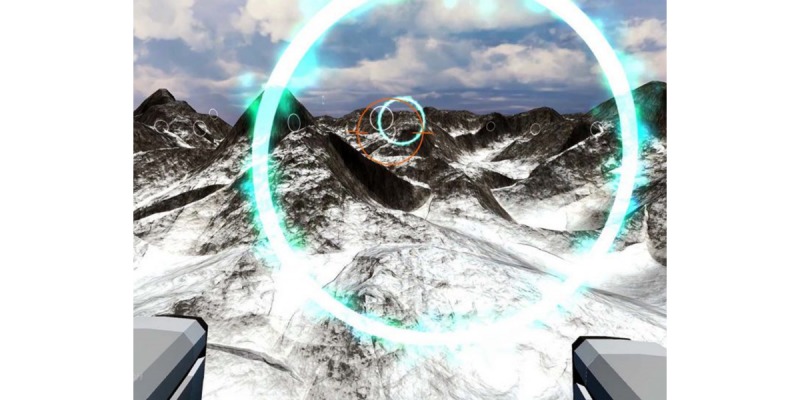
In-game screenshot of the participant field of view.

In a 5-minute familiarization session, participants were introduced to the VR in-game tasks. Afterward, eight electrodes were positioned on the recommended sensor locations for the *m. erector spinae* (neck extensors), *m. deltoideus pars clavicularis*, *m. rectus abdominus*, and *m. erector spinae* (lumbar region) as per the Surface EMG for Noninvasive Assessment of Muscles guidelines [39] (Figure 2). Participants were then asked to perform a maximum voluntary isometric contraction (MVIC) for each muscle [40]. Participants completed two consecutive flight sessions, approximately five minutes each, with an interim pause of 15 minutes. The goal of the game was for the participants to navigate their virtualized plane in a first-person view through all 63 rings. Computer software allowed the researchers to see the participant field of view (Figure 3). The speed of the vehicle and the horizon view were standardized to ensure maximal consistency in flight path, speed, and trajectory. During both sessions, muscle activity, heart rate, and device movements were continuously captured. Exertion levels were determined after each successful flight using the RPE scale. After the second flight, participants completed postsession questionnaires (SSQ, NASA-TLX, and PACES).

**Table 1:** Sociodemographic and anthropometric characteristics of study participants.

Variable	Finishers (n=25), mean (SD)	Motion sickness dropouts (n=6), mean (SD)
Age in years	24.16 (4.82)	25.00 (1.90)
Height (meters)	1.80 (0.63)	1.88 (0.08)
Weight (kg)	77.50 (8.49)	83.00 (6.10)
Body mass index (kg/m^2^)	23.80 (2.03)	23.36 (1.18)

### Outcome Measurements

The captured sEMG signal was smoothed using the root mean square algorithm, and the time interval was set to 300 milliseconds [41]. Afterward, the amplitude of the signal was normalized to the MVIC stack of the participant.

For the purposes of data analysis, a MATLAB script was programmed to provide a time sync of the Icaros device position along with the corresponding muscle activation and heart rate.

### Statistical Analysis

The captured data were exported from the MATLAB environment and statistically analyzed (SPSS Statistics 23, IBM Corp).

### Power

Power analysis indicated that for an estimated effect size of 0.6 with 80% power and 5% type I error, 25 participants were needed. Flight sessions that had to stop due to muscle pain or cybersickness symptoms were excluded from all data analysis except the SSQ score.

## Results

### Overview

Six participants had to stop the experiment due to signs of nausea or discomfort indicative of motion sickness. Another two participants stopped the VR flights because of muscle pain due to prior injuries. Post hoc analysis was computed based on a 5% type I error and a total of 25 participants.

### Muscle Activity During Flight Sessions

**Figure 4:**
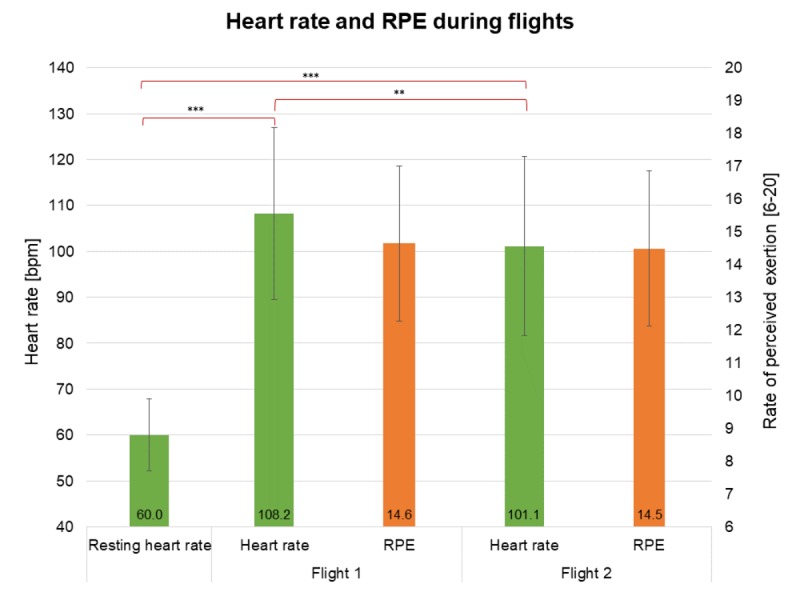
Average muscle activation during the two consecutive flights.

A paired sample mean *t* test resulted in significant differences between the two flights for the sEMG signal captured on the *m. erector spinae* muscles (neck extensors) of the participants (Figure 4). During the first flight, participants had significantly higher muscle activity (mean 24.21 [SD 11.47]) relative to the second flight (mean 20.60 [SD 10.57]), with *t*_24_=2.219, *P*=.04, and *d*=0.33. Achieved power was 35%.

### Heart Rate and Rate of Perceived Exertion During Flight Sessions

**Figure 5:**
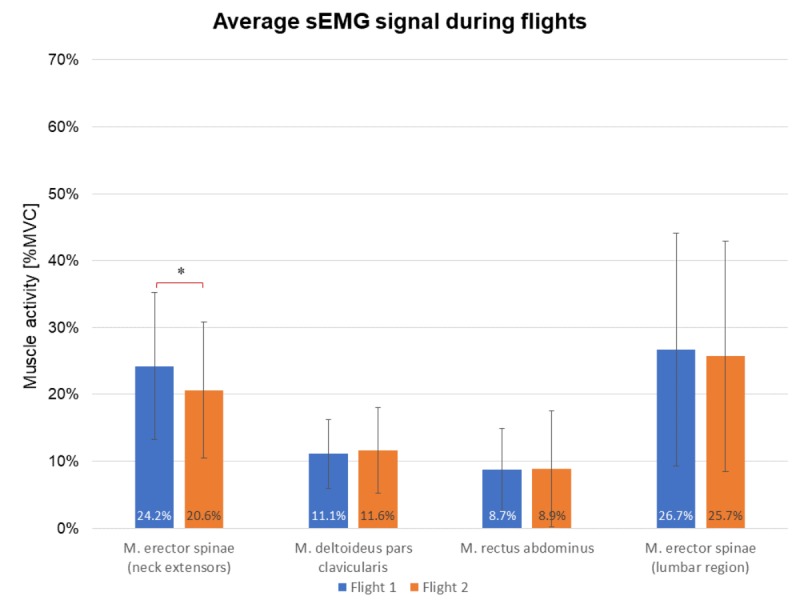
Heart rate and rate of perceived exertion during the two flights.

There were significant differences between resting heart rate and heart rate during the flights (Figure 5).

The one-way analysis of variance with repeated measures and a Greenhouse Geisser correction showed significant differences in the three heart rate measurements *F*_1.56,37.55_=161.29, *P*<.001, and *η* ²=0.87. A Bonferroni post hoc test indicated significant differences between the resting heart rate and the heart rate during flight 1 (*P*<.001, *d*=0.87; –48.18, 95% CI –56.51 to –39.86) and during flight 2 (*P*<.001, *d*=0.83; –41.07, 95% CI –49.51 to –32.64).

Average heart rate during flight 1 was significantly higher (*P*=.005, *d*=0.37; –7.11, 95% CI 1.98 to –12.23) than during flight 2.

Results from the paired sample *t* test showed no significant differences between participant RPE scores at the end of the first flight (mean 14.64 [SD 1.82]) and the second flight (mean 14.48 [SD 2.37]). Achieved power was 98%.

### Movements During Flight Sessions

**Figure 6:**
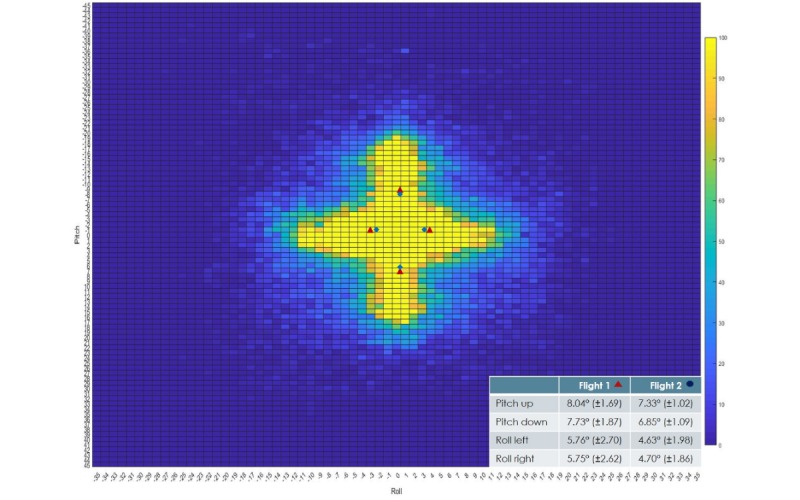
Heat plot of the Icaros device’s movements for both flights. Symbols depict the average positions throughout the flights on each axis.

Participants were able to steer pitch and roll by changing their body’s center of gravity. A paired *t* test showed that the average upward movements (Figure 6) on the pitch axis during flight 1 (mean 8.04° [SD 1.69]) were significantly larger, with *t*_24_=–2.370, *P*=.03, and *d*=0.51, than those during flight 2 (mean 7.33° [SD 1.02]). Achieved power was 69%.

Downward pitch movements also showed a significant difference, with *t*_24_=3.013, *P*=.06, and *d*=0.57 between flight 1 (mean 7.73° [SD 1.87]) and flight 2 (mean 6.85° [SD 1.09]). Achieved power was 78%.

The average degrees of roll were similar in both directions. Rolling left showed a significant difference, with *t*_24_=3.103, *P*=.05, and *d*=0.48 between flight 1 (mean 5.76° [SD 2.70]) and flight 2 (mean 4.63° [SD 1.98]). Achieved power was 63%.

Rolling right also showed significantly higher average degrees for flight 1 (mean 5.75° [SD 2.62]) than for flight 2 (mean 4.7° [SD 1.86]), with *t*_24_=–2.714, *P*=.012, and *d*=0.46. Achieved power was 60%.

### Questionnaires

Postsession questionnaires included the SSQ, NASA-TLX, and PACES. Table 2 illustrates the average values of the calculated dimensions and scores.

Levene test indicated unequal variances for all but the nausea score. Therefore, the degrees of freedom were adjusted from 29 to 5.26 for oculomotor, from 29 to 5.31 for disorientation, and from 29 to 5.35 for the total score. Due to the different sample sizes, a weighted effect size based on Hedges’ G formula replaced the Cohen *d* effect size.

The independent *t* test indicated a significantly higher score on all four SSQ subcategories. Participants who stopped the experiment due to motion sickness symptoms showed significantly higher oculomotor, disorientation, nausea, and total score values than the finishers (Table 2).

Participants found the sessions to be very physically demanding and exerted much effort when completing the session task. The task was not perceived as mentally demanding; its pace was not perceived as hurried or rushed (temporal demand). Participants felt like they achieved very good results (performance) and didn’t feel particularly insecure, discouraged, irritated, stressed, or annoyed (frustration). Despite the high physical demand scores and the moderate to vigorous RPE scores, participants rated the flights as highly enjoyable (PACES score 3.74 [SD 0.16]).

**Table 2:** Postsession questionnaire scores by dimension.

Questionnaire and dimension		Finishers	Motion sickness dropouts	Significance *P* value	Effect size g
**SSQ^a^**					
	Oculomotor	22.44 (15.85)	87.17 (48.11)	.02	2.62
	Disorientation	23.39 (29.72)	146.16 (82.93)	.01	2.80
	Nausea	27.86 (27.52)	124.02 (34.66)	<.001	3.24
	Total score	28.27 (21.51)	130.90 (56.67)	.006	3.35
**NASA-TLX^b^**					
	Mental demand	45.04 (24.61)	—^c^	—	—
	Physical demand	76.76 (15.73)	—	—	—
	Temporal demand	43.04 (18.73)	—	—	—
	Performance	74.88 (16.84)	—	—	—
	Effort	74.52 (10.98)	—	—	—
	Frustration	33.24 (24.78)	—	—	—
PACES^d^		3.74 (0.16)	—	—	—

^a^SSQ: Simulator Sickness Questionnaire.

^b^NASA-TLX: NASA Task load Index.

^c^Not available.

^d^PACES: Physical Activity Enjoyment Scale.

## Discussion

### Principal Findings

#### Muscle Activity

For isometric contractions, there is a good correlation between muscle activity and generated force [42]. The electrical signal measured on muscles is proportional to the muscle force, although there is debate about the linearity of this relationship [43]. Generally, though, MVIC reflects maximal muscle force. The MVIC normalized muscle activity signal can thus be used as a control mechanism on the effectiveness of muscle strengthening exercises. Depending on the generated muscle force, the effects can vary between mobilization and strength improvement with muscle growth [44].

Muscle activity during flights indicates the effects on muscle blood flow stimulation for all muscle groups. Kibler [45] notes that a minimum of 30% MVIC is required for effective strength endurance training; percentages below 30% provide mere muscle mobilization. In both flights, muscle activity for all assessed muscles ranged between 11% and 26% MVIC.

Dorsal muscle chain activity (neck extensors and lumbar region of *m. erector spinae*) shows higher levels of activation, with values reaching or crossing the 30% threshold in some cases. Dorsal muscle chain exercises (eg, stabilization exercise) could assist in alleviating low back pain in the lumbosacral area [28].

Participants position on the Icaros device resembles the well-established plank exercise. The only difference is the device’s shin holders, which provide additional support for users (Figure 1). This explains the rather low activation of the *m. rectus abdominus* and *m. deltoideus pars clavicularis* during both flights (Figure 4). Other studies reported EMG values of 44% to 65% on the rectus abdominus during the performance of plank exercises with unstable devices (such as the Swiss Ball) [29,46].

To achieve the most immersive experience, the virtual horizon was adjusted to a 45° angle, which required the participants to tilt their heads back. This position requires an almost constant isometric muscle contraction of the neck extensors. The neck extensors’ average muscle activation was relatively high compared with that of the *m. rectus abdominus*, the *m. deltoideus* *pars clavicularis*, and the *m. erector spinae* (lumbar region). In part, the high muscle activation was due to the extra weight (approx. 300 grams) of the head-mounted display. During flight 1, participants’ average muscle activity of the neck extensors was significantly higher than flight 2 (*P*=.04). Because of increased familiarization with the VR in-game elements, participants undertook fewer head movements during the second flight, which significantly lowered neck extensor muscle activation. The second flight did not show any other significant differences regarding muscle activity.

The similarity between the average muscle activations seems to indicate the potential reliability of the Icaros VR system. Being able to reproduce the physiological muscle activation in a consecutive flight suggests that familiarization effects do not reduce muscle activity straight away. Nevertheless, the long-term outcome of repetitive flights cannot be assessed based on this study’s design. The sample size consisted mainly of fit, young sport students whose athleticism distinguishes them from the general public.

Since the second flight was the same as the first one in terms of the virtualized in-game world and properties (eg, flying speed), differences between the two flights are attributable solely to participants’ movements.

The heat plot in Figure 6 shows the differences in the range of motion between the first and second flights. Standardizing the body balance point for all participants ensured that body height and weight would not bias muscle activity or device movements.

During the first flight, participants rolled by an average of 5.76° to the left and 5.75° to the right. Pitch movements reached 8.04° downward and 7.73° upward. Flight 2 resulted in a significantly smaller range of motion across all four directions, with effect size varying between 0.46 and 0.57. The smaller range of motion during the second flight can be attributed to familiarization with the in-game elements and device properties during the first flight.

Improved intermuscular coordination from reintroducing and sustaining body balance on the device after pitch and roll movements could also explain the significantly smaller range of motion. Interestingly, the significantly smaller range of motion did not correspond to significantly lower muscle activation during the second flight (see Figure 5). The measured muscle activation seems to be necessary for maintaining the plank position and coping with in-game goals. Compared with other exergaming systems, this represents a step forward. In some other fitness games, for instance, intended exercises can be avoided by simply shaking the remote controller quickly up and down.

Due to the mostly static position of the participants on the device, the effects on their cardiovascular system were expected to be low. Their resting heart rate (60.04 [SD 7.90] bpm) was significantly lower than their heart rate during the first (108.22 [SD 18.72] bpm) and the second (101.12 [SD 19.53] bpm) flights. Despite the significant differences between the two, the effect size was small to medium (*d*=0.37). The heart rate stayed within a lower aerobic intensity level, and the changes did not reflect a reduction in the intensity zone [47]. Because muscular activity showed no significant differences for all muscles but one, significant changes in heart rate should be attributed to psychological factors rather than to physiological ones. In his meta-analysis, Howard [48] investigated how excitement could potentially account as one factor for the success of VR rehabilitation programs. Furthermore, Bun et al [49] observed increased excitement during users’ first contact with VR systems. Such an aroused psychological state could further stimulate the sympathetic nervous system, increasing users’ heart rates.

Although heart rate alone is an insufficient metric for measuring energy expenditure and physiological effect [50], the observed heart rate values do seem to point to the positive effects of a low-intensity aerobic training zone. However, it must be borne in mind that the participants needed around 6 minutes per flight session, during which they mostly remained in a static isometric position that undercut the effects of aerobic cardiovascular training.

For muscle strength endurance training, long periods of muscle tension are connected with significantly higher myofibrillar protein synthesis [51]. However, long sessions of isometric exercises are also connected with high blood pressure levels, which are a risk factor for people with undiagnosed hypertension, heart disease, or other cardiovascular problems [52,53]. Such hypertensive participants can develop increased blood pressure and may need a longer period to recuperate than healthy individuals [54].

The reported RPE values of 14.56 (SD 2.09) for both flights indicate a moderate to vigorous activity level. The question of whether the perceived intensity level is due to physical arousal or due to cognitive workload can be further analyzed by looking at the reported NASA-TLX values, in which mental demand is lower than physical demand. Despite the perceived medium to high exertion levels, the reported enjoyment (PACES) during the VR sessions was high (3.74 [SD 0.18]), indicating that the VR system was fun to use. The perceived exertion, task workload, and enjoyment level are good indicators of a system’s potential to provide a good combination of fun and physical activity.

#### Motion Sickness and Cybersickness

Cybersickness can be described as a visually induced motion sickness that is common in immersive VR sessions [55]. Although the SSQ was not explicitly developed for assessing cybersickness, it is widely used in assessing the outcome of experience with VR environments [56-58]. The 6 participants with the strongest motion sickness symptoms had to stop the experiment. After the symptoms faded, they completed the accompanying questionnaire. They showed significantly higher scores on the SSQ than participants who were able to finish the flights (Table 2), with large effect sizes on all 4 subcategories.

The (forced) planking position on the Icaros was never before applied in a fully immersive VR system. It introduces high immersiveness by keeping the users on the transversal plane, simulating the in-game flying position. Changing of the visual stimuli as the device shifts on the axes is a unique feature of the system. Flights with the Icaros system seem to lead to relatively high SSQ scores in general [59]. Here, three properties come into play: the technical elements of the head-mounted display, the participants’ posture, and their postural sway.

The system used for our study does not represent the state of the art for head-mounted display technology, which can be found in devices such as Oculus Rift (Facebook Technologies LLC) and Vive Pro (HTC Corporation). Screen flickering caused by high latency and a low refresh rate (60 Hz) of systems like ours has been associated with symptoms of motion sickness [60,61]. Interestingly, it has been proposed that increased realism and immersion through improved optic stimulus may exacerbate motion sickness symptoms [55] as the internal focus is steered toward the sensory mismatches between the visual and vestibular systems.

Gallaghar et al [55] describes the vital role of the vestibular system in causing motion sickness symptoms. The most established cybersickness theories identify multisensory signal conflicts caused mainly by visual and vestibular signal mismatches as the root of motion sickness symptoms. On the Icaros device, posture and movement affect visual stimuli and perceived self-motion, which should lead to fewer visual-vestibular signal discrepancies.

Riccio and Stoffregen [62] propose that postural instability causes motion sickness symptoms. Smart et al [63] point out that postural sway could be related to higher perceived levels of visually induced motion sickness. On the Icaros device, the user’s body posture resembles the plank position but undergoes fast changes on pitch and roll axes, which could explain the perceived motion sickness. As users tried to maneuver through the rings, unpredictable changes in the device’s direction may have further influenced the perceived motion sickness [60]. Moreover, the vection produced by user head movements as they aimed for the next virtual ring may have also contributed to motion sickness [64,65].

### Limitations and Strengths

There are some limitations that warrant discussion. First, requirements regarding the physiological status of participants and the low fat-to-body ratio were very strict. While these requirements ensured high-quality and reliable EMG activation signals, they also restricted the transferability of the results to populations with different body compositions. Furthermore, the exclusive use of male participants for a consistent perception of cybersickness symptoms rendered the study unable to assess the physiological and psychological outcomes on female participants [32]. A third limitation was the assessment of training level intensity based on heart rate alone. Training heart rates can vary among individuals due to variances between actual maximal heart rates and the ones resulting from age-based formulas [50,66]. To eliminate the effects of these variances, we recommend the use of additional tools such as spirometry for future studies measuring physiological effort and energy expenditure.

This paper tries to investigate the combination of a fully immersive VR system with physical activity through a sport-science–based approach. To the best of our knowledge, this is the first qualitative study that examines the muscular training potential of an immersive VR-based full-body exergaming system.

We use established measuring procedures to identify muscular activity in terms of muscle innervation and potential training effects. Our approach points out future development steps of similar VR exergaming systems, assisting developers to optimize their efforts in promoting physical activity.

### Conclusion

The Icaros flight session appears to provide little to no cardiovascular benefit. Based on WHO guidelines for resistance training, muscle activity during the sessions occasionally met the lower threshold for effective muscle activation. The lower back’s muscle activation corresponds to plank variations with instability devices (eg, planks on exercise balls) [29].

By contrast, Icaros can provide improved muscle strength, especially for the dorsal muscle chain. Differentiated muscle activation can be achieved in virtualized worlds by, say, requiring the user to spend more time in a pitch down position, thereby shifting the body’s center of gravity on the pitch axis. But achieving the 30 minutes of daily cardiovascular activity recommended by the WHO requires dynamic instead of isometric movements.

Performing a plank position without external control of one’s body posture can result in hyperlordosis, an excessive extension of the lumbar region. Added stress on the visual and vestibular systems is associated with higher immersiveness but can lead to proprioceptive signals receiving less attention by the user. Spending time in flexed and awkward positions has been associated with low back pain syndrome [67]. It is crucial, therefore, that users’ movements do not amplify unwelcome body positions, especially during shifts in the body’s center of gravity.

Motivating the public to engage in physical activity is probably one of the most important and difficult tasks of the health sector. Gamification of physical exercise can help not only to motivate physical activity but also to promote social contact and interaction.

Future full-body and fully immersive concepts should focus on increasing dynamic muscle activation while considering user susceptibility to cybersickness and motion sickness. VR systems can give the public a refreshing and enjoyable form of physical activity.

In prevention and therapy, movement quality is a fundamental component of effective resistance training to benefit health. It is crucial, therefore, that future VR-based training systems follow the recommendations of sport and exercise science.

## Abbreviations

MVIC: maximum voluntary isometric contraction
NASA-TLX: NASA Task Load Index
PACES: Physical Activity Enjoyment Scale
RPE: rate of perceived exertion
sEMG: surface electromyography
SSQ: Simulator Sickness Questionnaire
VR: virtual reality
WHO: World Health Organization
